# Dehydration-responsive features of *Atrichum undulatum*

**DOI:** 10.1007/s10265-016-0836-x

**Published:** 2016-06-02

**Authors:** Ruoyang Hu, Lihong Xiao, Fang Bao, Xuedong Li, Yikun He

**Affiliations:** 1 School of Life Sciences, Capital Normal University, Beijing, 100048 People’s Republic of China; 2 Shanghai Center for Plant Stress Biology, Chinese Academy of Sciences, Shanghai, 201602 People’s Republic of China

**Keywords:** Basal land plants, Biological responses, Desiccation tolerance, Modern agriculture, Polytrichales, Ultrastructure

## Abstract

Drought is an increasingly important limitation on plant productivity worldwide. Understanding the mechanisms of drought tolerance in plants can lead to new strategies for developing drought-tolerant crops. Many moss species are able to survive desiccation—a more severe state of dehydration than drought. Research into the mechanisms and evolution of desiccation tolerance in basal land plants is of particular significance to both biology and agriculture. In this study, we conducted morphological, cytological, and physiological analyses of gametophytes of the highly desiccation-tolerant bryophyte *Atrichum* *undulatum* (Hedw.) P. Beauv during dehydration and rehydration. Our results suggested that the mechanisms underlying the dehydration–recovery cycle in *A. undulatum* gametophytes include maintenance of membrane stability, cellular structure protection, prevention of reactive oxygen species (ROS) generation, elimination of ROS, protection against ROS-induced damage, and repair of ROS-induced damage. Our data also indicate that this dehydration–recovery cycle consists not only of the physical removal and addition of water, but also involves a highly organized series of cytological, physiological, and biochemical changes. These attributes are similar to those reported for other drought- and desiccation-tolerant plant species. Our findings provide major insights into the mechanisms of dehydration-tolerance in the moss *A. undulatum*.

## Introduction

The natural habitats of plants are sometimes unfavorable, which greatly affects plant productivity. Among the various abiotic stresses limiting plant productivity, drought as a moderate dehydration state is of particular importance to modern agriculture. Consequently, elucidation of the mechanisms of drought tolerance can lead to new strategies for developing drought-tolerant crops. To survive and sustain growth under unsuitable conditions, various plants have evolved responses to drought (no bulk cytoplasmic water present, with approximately 0.30 g H_2_O per g dry weight, DW), desiccation (a more severe dehydration state than drought, in which the hydration shells of molecules are lost, with water content as low as 50 mg/g DW), and other stresses at multiple levels (Boyer 1982). The common mechanisms underlying these responses appear to be the accumulation of osmoprotectants, activation of reactive oxygen species (ROS) scavengers, and protection of membrane integrity (Mahajan and Tuteja 2005). Components involved in these processes, such as soluble sugars, glycine betaine, proline, malondialdehyde (MDA), glutathione reductase, superoxide dismutase (SOD), peroxide dismutase (POD) and glutathione (GSH), have been widely reported in organisms ranging from basal land plants to angiosperms (Reddy et al. 2004).

Bryophytes, the progenitors of terrestrial plants, do not have specific water-conducting tissue like that found in vascular plants (Cove et al. 1997). Many bryophytes can withstand rapid drying and are strongly tolerant to levels of drought that can be fatal to angiosperms. The results of several studies have suggested that the model desiccation-tolerant (DT) moss *Syntrichia ruralis* can survive after both slow and rapid desiccation (Fernández-Marín et al. 2011; Hamerlynck et al. 2000; Pressel and Duckett 2010). This moss has evolved a constitutive protection system to alleviate the damage caused by dehydration (Oliver et al. 2000, 2009). *Syntrichia caninervis* and *Bryum argenteum* are both classified as DT mosses, and their responses to desiccation have been widely studied (Gao et al. 2014, 2015; Li et al. 2014; Wu et al. 2012; Zheng et al. 2011).

*Atrichum undulatum* (Hedw.) P. Beauv. (Polytrichales), as the basal representative of the moss phylogenetic tree, is a desiccation-tolerant plant (Beckett et al. 2000). Recent studies by Beckett, Mayaba, and coauthors suggested that another *Atrichum* species, *Atrichum androgynum*, can withstand equilibrium at nearly 0 % relative humidity (RH), corresponding to a relative water content (RWC) of 4 %, with a series of physiological changes (Beckett and Hoddinott 1997; Guschina et al. 2002; Mayaba et al. 2001, 2002; Mayaba and Beckett 2003).

The advent of modern biotechnology has given rise to widely applied, high-throughput approaches to investigate the intrinsic mechanisms of desiccation tolerance. Nevertheless, it is still important to characterize the morphological, physiological, and cytological aspects of drought stress response in desiccation-tolerant plants. Such information, especially for moss species, is of fundamental significance. Although *A. undulatum* has been proven to be a desiccation-tolerant moss by Beckett, the morphological, physiological, and cytological changes that occur under natural drought conditions remain unclear. In this study, we examined *A. undulatum* at morphological, physiological, and cytological levels under simulated natural drought conditions to shed light on the evolution of dehydration responses in land plants. We hypothesized that during the dehydration–rehydration cycle, the mechanisms underlying maintenance of membrane stability, protection of cellular structure, defense against ROS generation, and elimination and repair of damage are operative in *A. undulatum*.

## Materials and methods

### Plant materials

Samples of the moss *A. undulatum* with mature capsules were collected from a shady, moist understory on Wuling Mountain, Hebei Province, China. Healthy capsules were selected and surface-sterilized as follows: (1) five 3-min rinses with sterilized water; (2) five rounds of sterilization for 5 s with 75 % ethanol and washing for 1 min with sterilized water; (3) sterilization with 0.05 % HgCl_2_ solution for 2 min; and (4) five 1-min washes with sterilized water. The capsules were transferred into sterilized water to prepare a spore suspension, which was inoculated onto the surface of Beneke’s medium containing 0.5 % (w/v) glucose (pH 5.8), and were cultivated 4 weeks under the following conditions: 25/20 °C day/night temperature, a 14-h/10-h light–dark photoperiod, illumination at 100 µmol photons m^−2^ s^−1^, and approximately 85 % relative humidity (RH). The *A. undulatum* cultures were then blended using a Tissue-Tearor (Bio Spec Products Inc., Bartlesville, OK, USA) and subcultured on Beneke’s medium containing 0.5 % (w/v) glucose for 2 weeks. Gametophores were induced during the second week. One-week-old gametophores were transferred to a plate containing Murashige-Skoog (MS) medium and 2 % (w/v) glucose (pH 5.8), and then incubated for an additional 2 weeks under the same light and temperature conditions mentioned above.

### Dehydration and recovery

To simulate natural dehydration conditions, 3-week-old whole *A. undulatum* plants (cultivated under approximately 85 % RH) were collected and dried on 20 pieces of filter paper in a controlled environment chamber under the following conditions (30 % RH, 25/20 °C day/night temperature, a 14-h/10-h light–dark cycle, and illumination at 100 µmol photons m^−2^ s^−1^).

After 3 days of dehydration, the moss gametophores were allowed to rehydrate by soaking in liquid MS medium supplemented with 2 % (w/v) glucose for 1 h, followed by transfer onto standard solid MS medium containing 2 % (w/v) glucose for recovery. Because the plants retained their green color during dehydration and early rehydration, survival was judged by the maintenance of green color by the moss tissues and the beginning of protonemal growth. Survival and death were calculated accurately from 24 to 72 h after rehydration.

### Measurement of water content and physiological responses

Hydrated and dehydrated tissues were collected at certain intervals and their fresh weights (FWs) were measured immediately. The DW was measured after drying for at least 48 h in a 65 °C oven. Water content (WC) was calculated using the formula WC = (FW − DW)/DW. At least five biological replicates were included for each time point.

Electrolyte leakage and MDA contents were examined to assess membrane stability, soluble sugars and proline contents were determined to evaluate cellular structure protection, and GSH, POD and SOD activities were determined to quantify antioxidant capacity (ROS elimination). Electrolyte leakage was measured using a DDBJ-350 electrical conductivity meter (INESA Scientific Instruments Co., Shanghai, China). The contents of MDA, soluble sugars, and proline, and the activities of GSH, POD, and SOD were measured using antioxidant detection kits (Nanjing Jiancheng Bioengineering Institute, Nanjing, China). Photosynthesis and respiration rates were detected using a LI-6400XT portable photosynthesis system (LI-COR, Lincoln, NE, USA). Chlorophyll fluorescence was measured using a IMAGING-PAM M-series chlorophyll fluorometer (Heinz Walz, Effeltrich, Germany).

### Transmission and scanning electron microscopy

Gametophytes of *A. undulatum* were fixed with 2 % glutaraldehyde in 0.1 M Sorensen’s phosphate buffer for 24 h, dried under vacuum for 2 h, post-fixed in 1 % (w/v) osmium tetroxide for 2 h, and then dehydrated for 30 min in an ethanol to acetone gradient. The samples were then divided into two portions. One portion was embedded overnight in Spurr’s low-viscosity resin. The embedded samples were dried at 40 °C for 2 days and then at 70 °C for 24 h. Sections were prepared with a Leica EM UC6 ultramicrotome (Leica Microsystems, Wetzlar, Germany) stained with 1 % aqueous uranyl acetate and 0.1 % aqueous lead citrate for 30 min each, and viewed under a Hitachi H7500 transmission electron microscope. The other sample portion was air-dried overnight in a laminar flow hood, sputter-coated with gold–palladium for 90 s at 2.2 kV, and examined under a Hitachi S4800 scanning electron microscope.

## Results

### *Atrichum undulatum* survival under dehydration

**Fig. 1:**
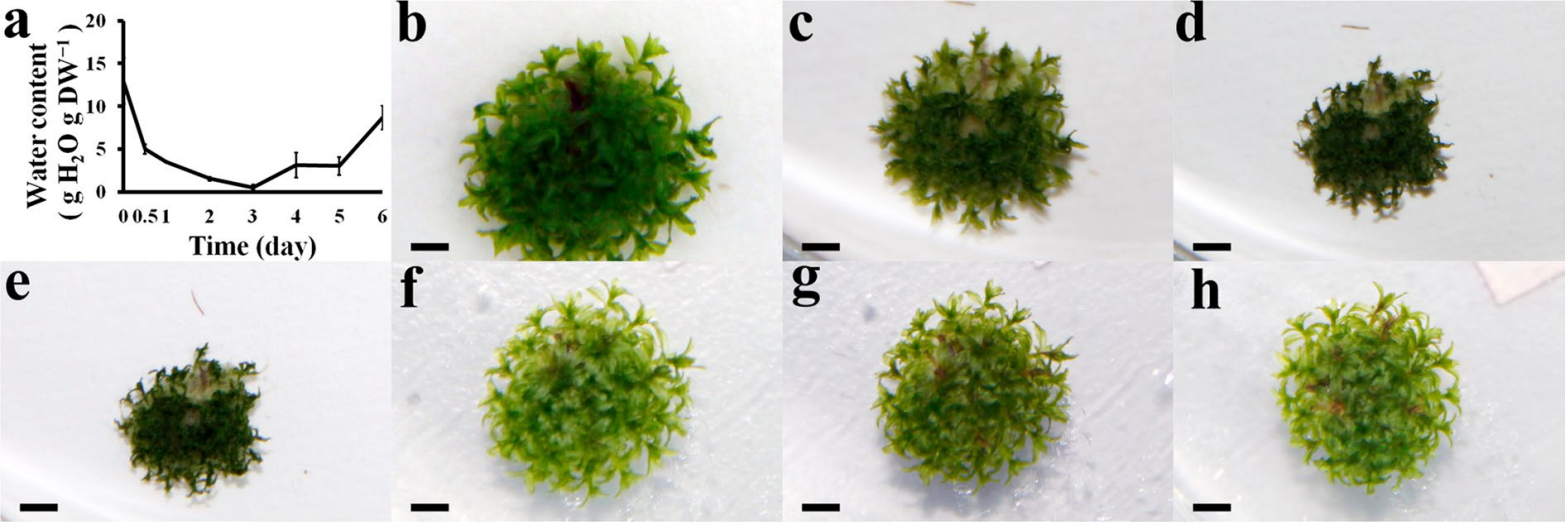
Water loss curve and phenotypes of *Atrichum undulatum* during drought and recovery. **a** Water loss curve; **b** hydrated control; **c**–**e** after dehydration for 1–3 days; **f**–**h** after recovery for 1–3 days. *Scale bars* 20 mm in **b**–**h**

To assess the dehydration tolerance of *A. undulatum* gametophores, we measured their WC during dehydration and subsequent recovery. During the first 24 h of dehydration, water loss was rapid (Fig. 1a). The WC reached the lowest point (0.54 ± 0.30 g H_2_O g^−1^ DW) on day 3 of dehydration. When water was resupplied, the dehydrated gametophores absorbed water rapidly. The morphology of mosses recovered to the hydrated control state after 3 d of rehydration. The WC had returned to 8.67 ± 1.41 g H_2_O g^−1^ DW, approximately two-thirds that of the hydrated control (12.96 ± 2.72 g H_2_O g^−1^ DW) level by day 6 (Fig. 1a), with more than 95 % of gametophores surviving.

### Morphological features

**Fig. 2:**
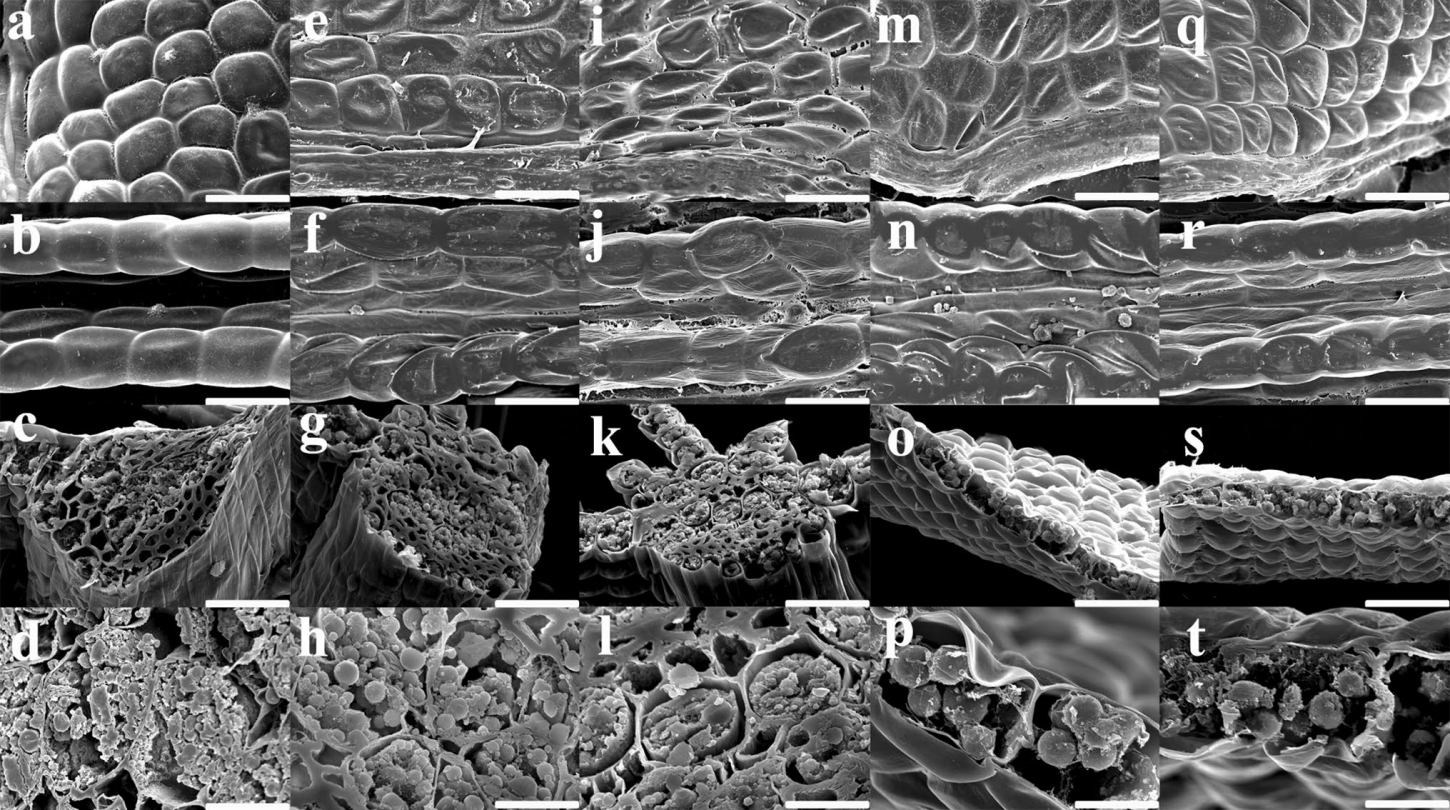
Morphological features of *Atrichum undulatum* leaves during dehydration and recovery. **a**–**d** Hydrated control; **e**–**h**, after 1 day of dehydration; **i**–**l** after 3 days of dehydration; **m**–**p** after 1 day of recovery; and **q**–**t**, after 3 days of recovery. **a**, **e**, **i**, **m** and **q** show leaf surfaces and **b**, **f**, **j**, **n** and **r** show leaf midribs. **c**, **g** and **k** are midrib sections, and **o** and **s** are leaves. Magnified views of sections are shown in **d**, **h**, **l**, **p** and **t**. *Scale bars* 30 µm in **a**, **b**, **e**, **f**, **i**, **j**, **m**, **n**, **q** and **r**, 50 µm in **c**, **g**, **k**, **o** and **s**, and 10 µm in **d**, **h**, **l**, **p** and **t**

For morphological characterizations, hydrated *A. undulatum* gametophores were dried for 3 days and then allowed to recover for an additional 3 days as assessed by a water loss curve and morphological observations (Figs. 1, 2). The mosses were severely stressed by 3 days of dehydration. During dehydration, the hydrated *A. undulatum* gametophytes (Fig. 1b) gradually turned dark green (Fig. 1c–e). Leaf shrinking and curling began at the leaf apex and gradually spread downwards (Fig. 1c–e). At the beginning of dehydration (Fig. 2e–h), shrunken cells first appeared at the leaf edges, but cells at the midribs were not visibly different from those in the hydrated controls (Fig. 2a–d). Under continued dehydration, cells at the leaf edges collapsed and plasmolysis occurred at the midribs; however, cell structures remained intact (Fig. 2i–l).

During recovery, the moss gametophytes rapidly absorbed water. The shrunken leaves expanded and the dark-green color disappeared on day 1 of rehydration; only 3.5 % of tender tissues were dead on day 3 of rehydration (Fig. 1f–h). On day 1 of rehydration, swelling of cells due to water absorption proceeded rapidly from the midribs to leaf edges (Fig. 2m–p). By day 3 of rehydration, almost all of the cells in the midribs and leaf edges had fully recovered (Fig. 2q–t).

### Cell ultrastructure

**Fig. 3:**
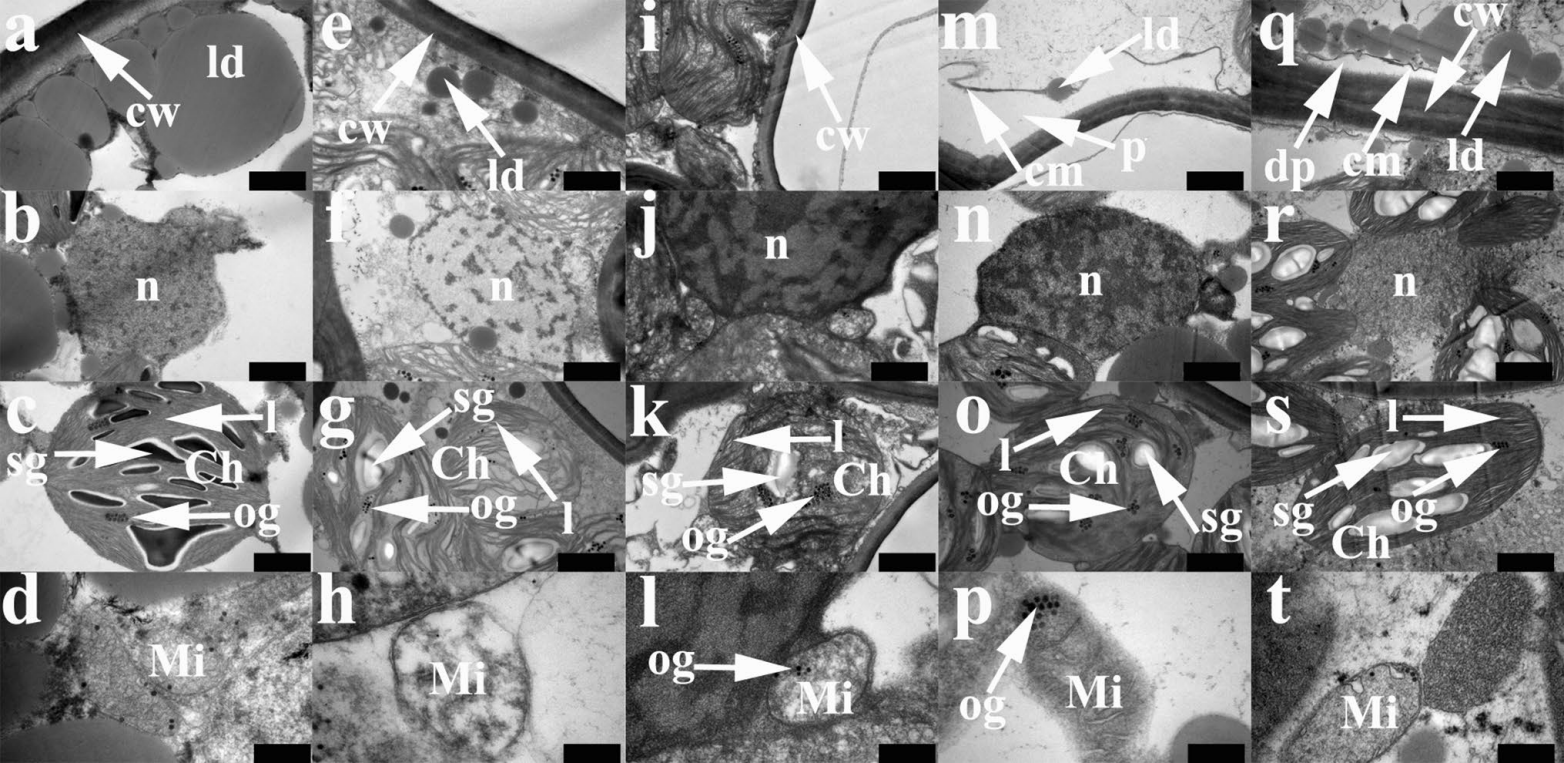
Cytological features of *Atrichum undulatum* during dehydration and recovery. **a**–**d** Hydrated controls; **e**–**h** after 1 day of dehydration; **i**–**l** after 3 days of dehydration; **m**–**p** after 1 day of recovery; **q**–**t** after 3 days of recovery. **a**, **e**, **i**, **m** and **q** show cell walls and membranes; **b**, **f**, **j**, **n** and **r** show nuclear structure; **c**, **g**, **k**, **o** and **s** are chloroplasts; and **d**, **h**, **l**, **p** and **t** are mitochondria. *cw* cell wall, *ld* lipid drop, *cm* cell membrane, *p* plasmolysis, *dp* deplasmolysis, *n* nucleus, *Ch* chloroplast, *l* lamellae system, *sg* starch granule, *og* osmiophilic granule, *Mi* mitochondria. *Scale bars* 1 µm in **a**–**c**, **e**–**g**, **i**–**k**, **m**–**o**, **q** and **s**, 2 µm in **r**, and 200 nm in **d**, **h**, **l**, **p** and **t**

To examine moss responses to dehydration and recovery in detail, we observed the cell ultrastructure (Fig. 3). Under dehydration stress conditions, *A. undulatum* protoplasts shriveled and cell walls gradually became depressed. Lipid droplets decomposed during dehydration (Fig. 3a, e, i). Plasmolysis occurred at the beginning of rehydration and deplasmolysis on day 3 of rehydration (Fig. 3m, q).

During dehydration, the chromatin in the nucleus condensed and adhered to the nuclear membrane (Fig. 3b, f, j). Chromatin remained in its condensed state during the early stage of rehydration, but relaxed as rehydration progressed (Fig. 3n, r).

Chloroplasts in hydrated cells of *A. undulatum* were generally ball- or lens-shaped, and starch granules in stroma and thylakoid systems were clearly visible (Fig. 3c). The chloroplast envelope was still present after dehydration, but grana and stroma lamellae became disorganized and swollen before disappearing. Starch granules became smaller, and osmiophilic globules formed (Fig. 3g, k). During recovery, thylakoid grana and their connecting lamellae were observable, starch granules re-accumulated, and the size and quantity of osmiophilic granules decreased (Fig. 3o, s).

Dehydration caused mitochondria to swell in *A. undulatum*; their inner membranes vanished, especially the cristae, and osmiophilic globules similar to those observed in chloroplasts became visible (Fig. 3d, h, l). In the fully recovered gametophores, the mitochondrial cristae membranes and granular matrix were restored and the osmiophilic granules disappeared (Fig. 3p, t).

### Physiological responses

**Fig. 4:**
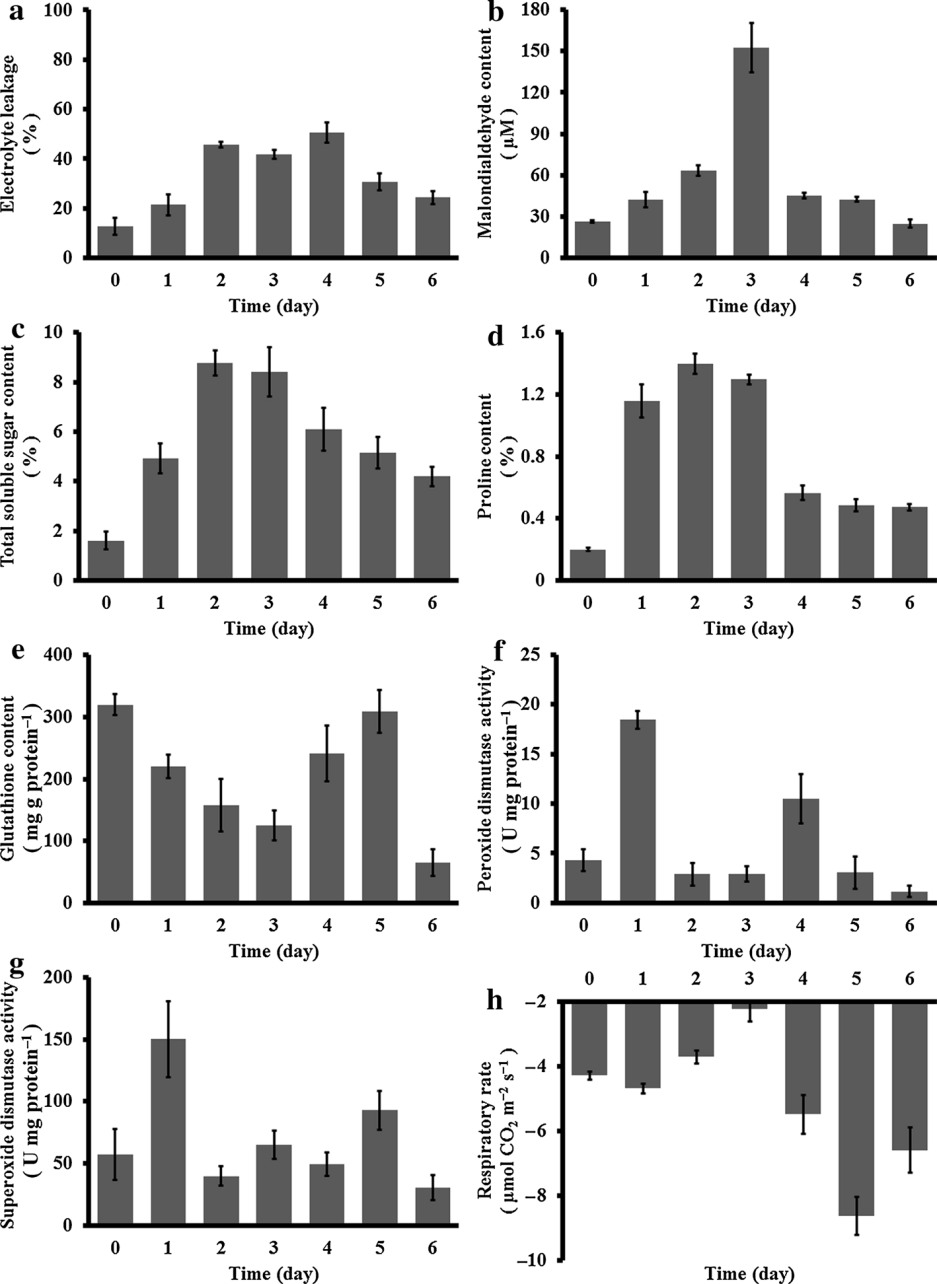
Physiological responses of *Atrichum undulatum* during dehydration and recovery. Measured parameters were electrolyte leakage (**a**), malondialdehyde content (**b**), total soluble sugar content (**c**), proline content (**d**), glutathione content (**e**), activity of peroxide dismutase (**f**), activity of superoxide dismutase (**g**) and respiratory rate (**h**). X-axis shows treatment time, corresponding to hydrated control (*0*), 1–3 days of dehydration (*1*–*3*) and 1–3 days of recovery (*4*–*6*). All measurements are based on three independent experimental and five technical replicates

Electrolyte leakage, a common indicator of cell membrane stability, was elevated in *A. undulatum* gametophores at the beginning of dehydration and remained constant after day 1 of dehydration. During rehydration, electrolyte leakage rose slightly during the first 24 h and then dropped (Fig. 4a). The level of MDA, which is an indicator of cell membrane damage, increased slowly during the first 2 d of dehydration, but increased markedly under severe dehydration (152.51 ± 18.12 μM) to sevenfold that of the hydrated control (26.38 ± 0.61 μM). After 3 days of rehydration, the MDA levels returned to 24.89 ± 3.14 μM (Fig. 4b).

The total soluble sugars content (TSS) was significantly elevated in *A. undulatum* under moderate dehydration. The TSS increased to its peak level (8.78 ± 0.50 %) after 3 days of dehydration and decreased only slightly to 4.19 ± 0.40 % by day 3 of rehydration (Fig. 4c). The proline content was initially very low (0.20 ± 0.01 %), but peaked rapidly after 1 days of dehydration and then remained steady (1.40 ± 0.06 %). Within 24 h of water restoration, the proline level dropped (0.57 ± 0.05 %) as quickly as it had increased upon dehydration, and remained at that level during rehydration (Fig. 4d). The proline level after 3 days of rehydration (0.47 ± 0.02 %) was still significantly higher than that of the hydrated control (0.20 ± 0.01 %).

The GSH content steadily decreased in *A. undulatum* under dehydration (from 319.99 ± 17.28 to 125.38 ± 24.03 mg/g prot.). During rehydration, the GSH content first increased to (309.24 ± 34.74 mg/g prot.) but eventually dropped to 64.64 ± 21.40 mg/g prot. (Fig. 4e). The POD and SOD activities showed similar trends, with maximum values on day 1 of dehydration (18.45 ± 0.91 U/mg prot. for POD and 150.19 ± 30.58 U/mg prot. for SOD) and then falling. During recovery, POD activity first increased and then decreased to 1.12 ± 0.57 U/mg prot., similar to the original level (4.30 ± 1.07 U/mg prot.), whereas SOD activity fluctuated before returning to 30.65 ± 10.01 U/mg prot., similar to that in the hydrated control (57.16 ± 20.76 U/mg prot.) (Figure 4f, g).

The respiratory rate fell by half in severely dried *A. undulatum*, from −4.29 ± 0.13 to −2.24 ± 0.39 μmol CO_2_ m^−2^ s^−1^. During recovery, the respiratory rate increased twofold (−8.63 ± 0.58 μmol CO_2_ m^−2^ s^−1^) compared with that in the hydrated control (−4.29 ± 0.13 μmol CO_2_ m^−2^ s^−1^) and eventually reached −6.60 ± 0.71 μmol CO_2_ m^−2^ s^−1^ (Fig. 4h).

### Effects on photosynthesis

**Fig. 5:**
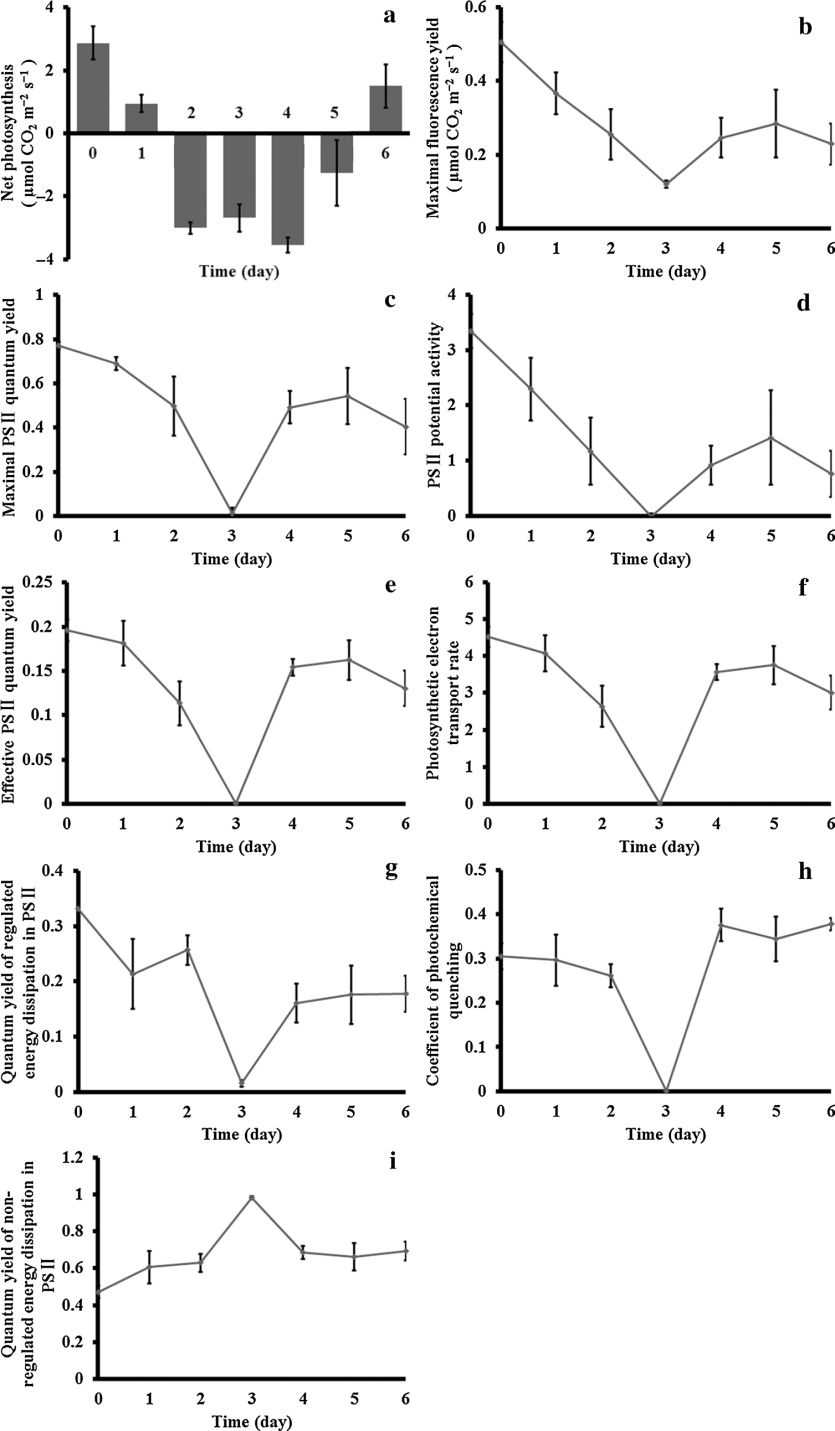
Changes in chlorophyll fluorescence and photosystem activity in response to dehydration and recovery in *Atrichum undulatum*. Measured parameters were net photosynthesis (**a**), maximal fluorescence yield (**b**), maximal PSII quantum yield (**c**), PSII potential activity (**d**), effective PSII quantum yield (**e**), photosynthetic electron transport rate (**f**), quantum yield of regulated energy dissipation in PSII (**g**), coefficient of photochemical quenching (**h**), and quantum yield of non-regulated energy dissipation in PSII (**i**). Horizontal axes show treatment time, corresponding to hydrated control (*0*), 1–3 days of dehydration (*1*–*3*) and 1–3 days of recovery (*4*–*6*). All measurements are based on three independent experimental and five technical replicates

Net photosynthesis (Pn), which represents the efficiency of converting light energy into chemical energy in plants, is very sensitive to stress. During the first day of dehydration, the Pn of *A. undulatum* dropped by about 50 % (from 2.87 ± 0.53 to 0.95 ± 0.28 μmol CO_2_ m^−2^ s^−1^), dropped further to −3.00 ± 0.19 μmol CO_2_ m^−2^ s^−1^ on day 2 of dehydration, and remained around that level until day 2 of recovery (−1.26 ± 1.04 μmol CO_2_ m^−2^ s^−1^). The Pn returned to 1.50 ± 0.69 μmol CO_2_ m^−2^ s^−1^, the same level as that in the hydrated control, on day 3 of rehydration (Fig. 5a). Maximal fluorescence yield (F_m_) declined incrementally under dehydration (from 0.51 ± 0.05 to 0.12 ± 0.01 μmol CO_2_ m^−2^ s^−1^) and returned to 0.28 ± 0.09 μmol CO_2_ m^−2^ s^−1^ during recovery (Fig. 5b). The maximal photosystem II (PSII) quantum yield (F_v_/F_m_), PSII potential activity (F_v_/F_0_), effective PSII quantum yield [Y(PSII)], photosynthetic electron transport rate (ETR), quantum yield of regulated energy dissipation in PSII [Y(NPQ)], and the coefficient of photochemical quenching (qP) showed similar trends during dehydration: a downward linear trend coupled with water loss. When the dried gametophores began to recover, Y(PSII), Y(NPQ), and qP rose steadily and peaked, while F_v_/F_m_, F_v_/F_0_, and ETR recovered to peak on day 2 of rehydration then declined slightly (Fig. 5c–h). The quantum yield of non-regulated energy dissipation in PSII [Y(NO)] showed an opposite trend to that observed for Y(NPQ), eventually reaching its highest value under dehydration and then gradually declining to the original level during rehydration (Fig. 5i).

## Discussion

Tolerance to cellular dehydration in bryophytes is probably an ancient characteristic of land plants. Although bryophytes do not have specific water-conducting tissue like that found in vascular plants (Cove et al. 1997), some have specialized water-conducting internal cells (Ligrone et al. 2002). This would have been an important feature to colonize land. In the genus Physcomitrella, a family of genes that regulate the development of hydroids—internal specialized water-conducting cells, has been identified (Xu et al. 2014). Most mosses can survive under −20 to −40 MPa for short periods, which far exceeds the range tolerated by most crop plants (−1.5 to −3 MPa) (Proctor and Pence 2002). Some mosses such as *S.* *ruralis* can survive at −540 MPa (equilibrated to 2–4 % RH using silica gel) (Oliver et al. 1993). This species shows excellent desiccation tolerance, and can recover from the dried state in several minutes (Proctor 2001). The moss species *Physcomitrella patens* has been widely used as an experimental model. Although its drought tolerance is thought to be induced by desiccation, *P. patens* is still more drought tolerant than most angiosperms and can resist slow desiccation (Greenwood and Stark 2014). Hamerlynck et al. (2000) showed that most dehydration-tolerant mosses, for example, *S. ruralis*, can alter gametophore structure to control water loss during dehydration, and so changes in surface reflectance can serve as a proxy measurement of water content. Our results indicate that the same changes occurred in *A. undulatum* during dehydration (Fig. 1). As reported by Beckett et al. (2000), *A. undulatum* can withstand equilibrium over silica gel for 24 h to a final RWC of 0.02. In comparison, our results imply that *A. undulatum* can tolerate an initial water loss of 90 %, thus displaying a level of desiccation tolerance intermediate between those of *S. ruralis* and *P. patens*.

Maintenance of membrane stability is critical for plant survival under environment stress. We observed that the curled leaves, plasmolysis, and increased electrolyte leakage during dehydration reversed during rehydration. These observations suggest that drought stress-induced membrane damage in *A. undulatum* gametophore cells is neither severe nor irreparable. The condensed state of chromatin and increased cytoplasmic viscosity at the beginning of dehydration (Figs. 1, 2) probably reflected the needs for survival in *A. undulatum*. These phenomena are responsible for two critical and coincident processes: preservation of cellular structure and protection against ROS damage. A reduction in cellular volume increases the chance of interactions between protoplasmic components such as proteins and lipids, accelerates the denaturation of components or membranes due to ROS, and increases lipid peroxidation, chlorophyll degradation, and DNA damage (Seel et al. 1992).

Soluble sugars are highly sensitive to environmental stress; they allow the membrane surface to remain preferentially hydrated, replace water in the hydration shell, and prevent membrane fusion. The absence of these solutes can cause membrane fusion, leading to a phase transition into the gel phase (Hoekstra et al. 2001). Water deficit causes ribosomes to shift from polymeric to monomeric forms, thereby affecting protein synthesis (Hsiao 1970) and leading to proline accumulation during dehydration. Proline may reduce stress-induced cellular acidification or promote oxidative respiration to provide the energy needed for recovery (Hare and Cress 1997). Consequently, the significant increases in TSS in *A. undulatum* during dehydration and the maintenance of high TSS at high levels during recovery (Fig. 4) provide an additional explanation for the strong dehydration tolerance of this moss.

In plants, ROS such as peroxide (H_2_O_2_), superoxide (O_2_^−^), and hydroxyl radicals (HO^**·**^), are major threats to plant cell survival under a variety of environmental stresses. Glutathione transferase (GT), peroxidase (GTpx), and reductase (GR) along with SOD and POD constitute an enzymatic antioxidant system that regulates oxygen toxicity. The activities of GT, GTpx, and GR are well correlated with the GSH/glutathione disulfide (GSSG) redox reaction, while GSH directly and indirectly controls ROS concentrations by removing H_2_O_2_, lipid peroxides, and methylglyoxal. Oxidation of GSH is responsible for low GSH to GSSG ratios during ROS detoxification (Szalai et al. 2009). The observed fluctuations in GSH activity during *A. undulatum* recovery (Fig. 4) may have been due to membrane damage and ROS toxicity. Peroxidases use a variety of electron donors to reduce H_2_O_2_. Superoxide dismutases are abundant in aerobic cells, which are dependent on their activity, and their active sites may contain Cu, Zn, Mn, Fe, or even Ni. The Cu/Zn SODs are found in the cytosols of eukaryotic cells, in the periplasms of gram-negative bacteria, and in the plastids of plants, while MnSOD and FeSOD are found in the matrices of mitochondria and chloroplasts, respectively. These enzymes maintain a steady-state level of cellular O_2_^−^ (Scalet et al. 1995; Smirnoff and Colombé 1988; Wang et al. 2003). Oxygen (O_2_), which is used in respiration and photosynthesis, has many toxic, cell-damaging effects when present at excess concentrations. Highly reactive metabolic products of O_2_ inactivate cellular enzymes, damage DNA, and destroy lipid membranes (Cadenas 1989). The HO^**·**^ radical is an extraordinarily powerful oxidant that binds to phospholipid membranes and causes the polar lipid fraction to decrease. The presence of MDA, which is a product of lipid peroxidation, is indicative of membrane deterioration (Stewart and Bewley 1980). The results of this and other studies on these antioxidants suggest that most land plants, from mosses to angiosperms, share similar enzymatic and molecular antioxidant mechanisms to eliminate deleterious ROS.

The photosystem is the most important cellular component for plant survival, growth, and development. Under drought conditions, light increases damage to plant tissues, especially the photosynthetic systems. Although the chloroplast envelope in *A. undulatum* barely differs between the dehydrated and rehydrated state, dehydration leads to disorganization of the lamellae systems and depletion of starch granules (Fig. 3). Similar changes were shown to occur in desiccated *Polytrichum formosum* (Proctor et al. 2007) and ABA-treated *P. patens* (Nagao et al. 2005). Previous studies have shown that chlorophyll fluorescence is almost totally suppressed during dehydration in bryophytes (Heber et al. 2001; Pressel et al. 2006; Proctor and Pence 2002). Consistent with those findings, chlorophyll fluorescence in *A. undulatum* was extremely low during dehydration (Fig. 5). Water deficit reduces the area available for CO_2_ uptake, resulting in a lower Pn. In *A. undulatum*, F_m_, F_v_/F_m_, and F_v_/F_0_ were constrained during dehydration, as were Y(PSII), ETR (E), and Y(NPQ). These results indicate that dehydration stress significantly affected PSII function in *A. undulatum*. In other words, the absorbed light energy was effectively dissipated as heat, and so it did not generate potentially damaging reactions. Thermal energy dissipation mediated by the xanthophyll cycle plays a significant role in photoprotection (Deltoro et al. 1998; Demmig-Adams and Adams 1992; Heber et al. 2006), and photoprotective mechanisms can minimize light damage (Logan 2008). Y(NO) represents both photochemical energy conversion and protective regulatory mechanisms efficiency. qP is based on the “puddle model” of PSII, in which the antennae of individual PSII reaction centers are connected. Consequently, energy can be transferred with high probability from closed reaction centers to neighboring open ones. The observed changes in both of these parameters indicate that *A. undulatum* was challenged by incident radiation on day 3 of dehydration, but all of the chlorophyll fluorescence parameters recovered quickly upon rehydration (Fig. 5). Similar results were reported for *P. formosum*, in which the chlorophyll-fluorescence parameters returned to predesiccation levels in 1–2 days (Pressel et al. 2006). This recovery may occur within hours in some other desiccation-tolerance mosses, for example, *S. ruralis* (Proctor and Pence 2002). The difference in recovery time may be related to the time needed for cytoskeleton reassembly (Pressel et al. 2006). In general, our results indicate that the photosynthetic system in *A. undulatum* was severely affected by dehydration and likely needed a long time to recover.

Taken together, our results show that maintenance of membrane stability, protection of cellular structure, defense against ROS generation, and elimination and repair of ROS-induced damage occur during the dehydration–recovery cycle in *A. undulatum*. Our integrated analysis of morphological, ultra-structural, and physiological features revealed that *A. undulatum* gametophores have strong abilities to maintain membrane stability and protect against ROS generation and ROS-induced damage in their responses to dehydration and recovery. Our results also suggest that the dehydration–recovery cycle does not merely entail the physical removal and addition of water, but also involves a highly organized series of cytological, physiological and biochemical changes, similar to previous findings in other drought- and desiccation-tolerant species (Proctor et al. 2007). Our findings provide major insights into the mechanisms of dehydration stress in this moss species, and should inform strategies for drought-tolerant crop development.
